# Highly efficient single-stack hybrid cool white OLED utilizing blue thermally activated delayed fluorescent and yellow phosphorescent emitters

**DOI:** 10.1038/s41598-018-34593-3

**Published:** 2018-11-02

**Authors:** Gyeong Woo Kim, Hyeong Woo Bae, Raju Lampande, Ik Jang Ko, Jin Hwan Park, Chae Young Lee, Jang Hyuk Kwon

**Affiliations:** Department of Information Display, Kyung Hee University, Dongdaemoon-gu, Seoul 130-701 Republic of Korea

## Abstract

Highly efficient single-stack hybrid cool white organic light-emitting diodes (OLEDs) having blue-yellow-blue multiple emitting layers (EMLs) are designed and constructed by utilizing blue thermally activated delayed fluorescent (TADF) and yellow phosphorescent emitters. The out-coupling efficiencies of yellow and blue emissions are maximized by tuning the ITO and total device thickness that satisfies both of antinode positions for yellow and blue emissions in a limited multiple EML thickness. To obtain a cool white emission, the exciton formation ratio in the blue-yellow-blue multiple EML system is controlled by manipulating the recombination zone through charge conductivity variation of host medium in the blue TADF EML. The resulting device exhibits cool white emission with very high maximum external quantum efficiency of 23.1% and CIE color coordinates of (0.324, 0.337). We anticipate that the studied approach will raise the viability of single-stack hybrid cool white OLEDs for high performance display applications.

## Introduction

White organic light-emitting diode (WOLED) is a core technology for large-area active matrix OLED (AMOLED) displays and a promising candidate for next generation lighting applications^1,2^. Recently, the importance of WOLED is increasing for large size OLED TV displays. OLED TV is successfully commercialized by adopting the OLED panel comprising WOLED with fluorescent blue and phosphorescent yellow green units in two-tandem configuration and red, green and blue color filters^3–5^. Indeed, in the cool WOLEDs (CWOLEDs), hybrid system utilizing fluorescent blue and phosphorescent green and red or yellow emitters are used to secure sufficient device lifetime because highly stable blue phosphorescent emitter has not been developed so far^6,7^. Generally, hybrid CWOLED structures are especially divided into single-stack and tandem configurations^8–12^. Among them, only three-stack tandem structure is adopted for display applications due to its high efficiency and long device life. In this tandem structure, two blue emitting layers (EML) are used to compensate low intensity of blue fluorescence, and thereby highly efficient cool white emission and long device life can be achieved. However, such a complicate structure not only increases manufacturing cost and driving voltage but also degrades viewing angle characteristics. On the other hand, single-stack hybrid WOLED has more simple structure compared to that of tandem structure. Hence, the device manufacturing cost can be reduced and it also provides much better viewing angle characteristics. However, low efficiency is the most challenging issue in single-stack hybrid WOLEDs.

**Figure 1:**
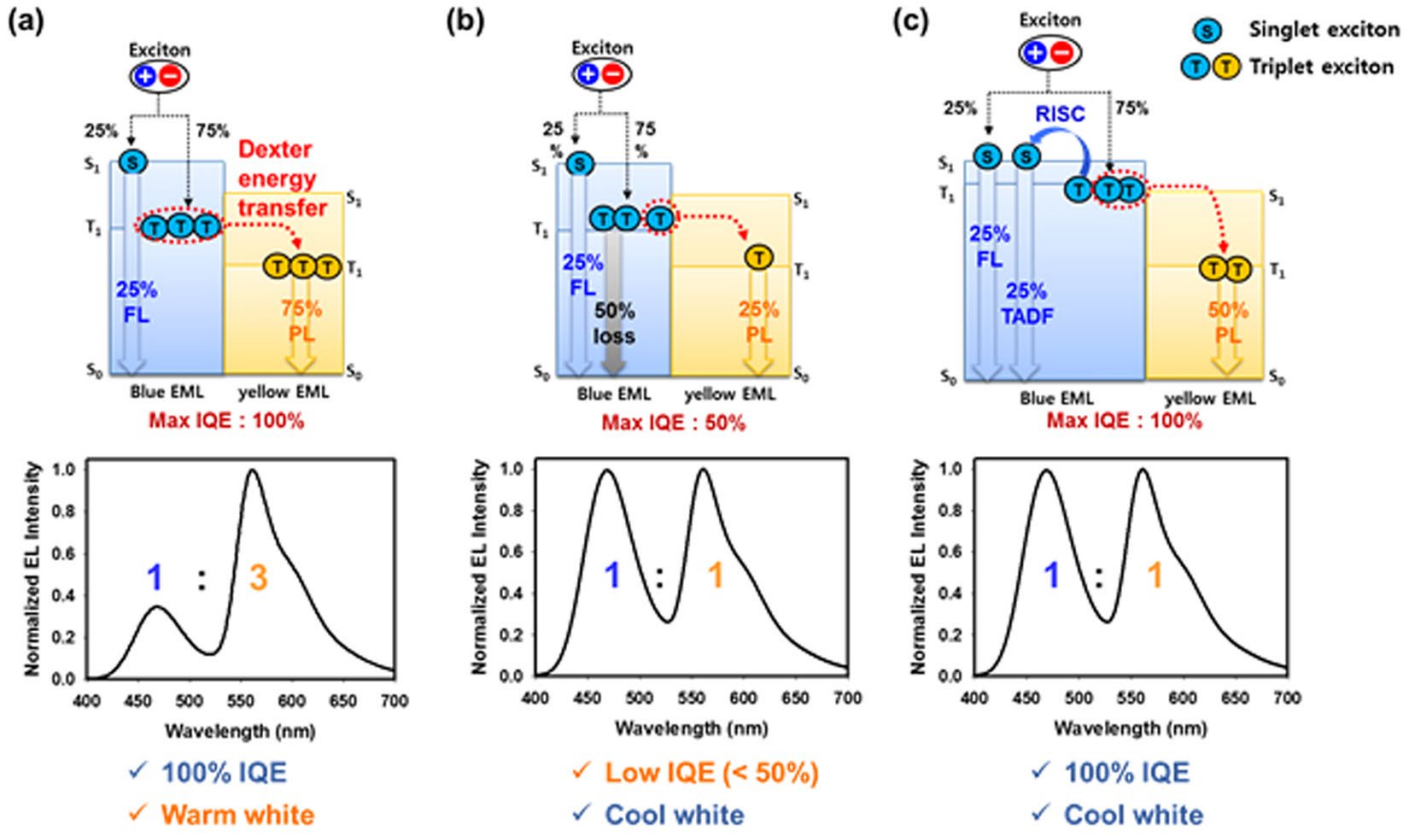
Schematic representations of exciton dynamics in fluorescent blue (**a**,**b**), TADF blue (**c**) phosphorescent yellow hybrid multiple EML system and their resulting EL spectra.

In the single-stack hybrid CWOLED, 100% internal quantum efficiency (IQE) can be achieved if all blue triplet excitons are converted to yellow triplet excitons via Dexter energy transfer as shown in Fig. 1(a). However, in that case, yellow emission is three times higher than the blue emission; therefore the resulting emission would be warm white. Thus, to achieve cool white emission, two-thirds of blue triplet excitons should be lost through non-radiative decay as shown in Fig. 1b, and that is the reason of low efficiency and the inevitable limitation of single-stack hybrid system using conventional blue fluorescent emitter. The most effective way to solve this issue is by introducing blue TADF emitter instead of the conventional blue fluorescent emitter. As shown in Fig. 1c, all electrically generated blue triplet excitons can be harvested through reverse intersystem crossing (RISC) from blue EML and Dexter energy transfer from yellow EML. Hence, 100% IQE and cool white emission can be realized at the same time. Nevertheless, very few papers on single-stack cool white OLEDs with blue TADF emitter have been reported so far^13,14^.

In this work, we investigate a device design method to achieve high efficiency and cool white emission from single-stack hybrid CWOLED. The blue-yellow-blue multiple EML structure is employed to harvest all electrically generated excitons and to balance the intensities of blue and yellow emissions. To maximize out-coupling efficiencies of yellow and blue emissions, the ITO and total device thickness that satisfy both of antinode positions for yellow and blue emission in the limited multiple EML thickness are considered. Also, the exciton formation ratio in the blue-yellow-blue multiple EML system is tuned by controlling recombination zone via charge conductivity variation of host materials in the blue TADF EML. By utilizing the optimum device thickness and the multiple EML system, very high external quantum efficiency (EQE) of 23.1% and cool white emission with CIE color coordinate of (0.324, 0.337) are achieved in single-stack hybrid CWOLED.

## Result and Discussion

### Material selection for white emission

**Figure 2:**
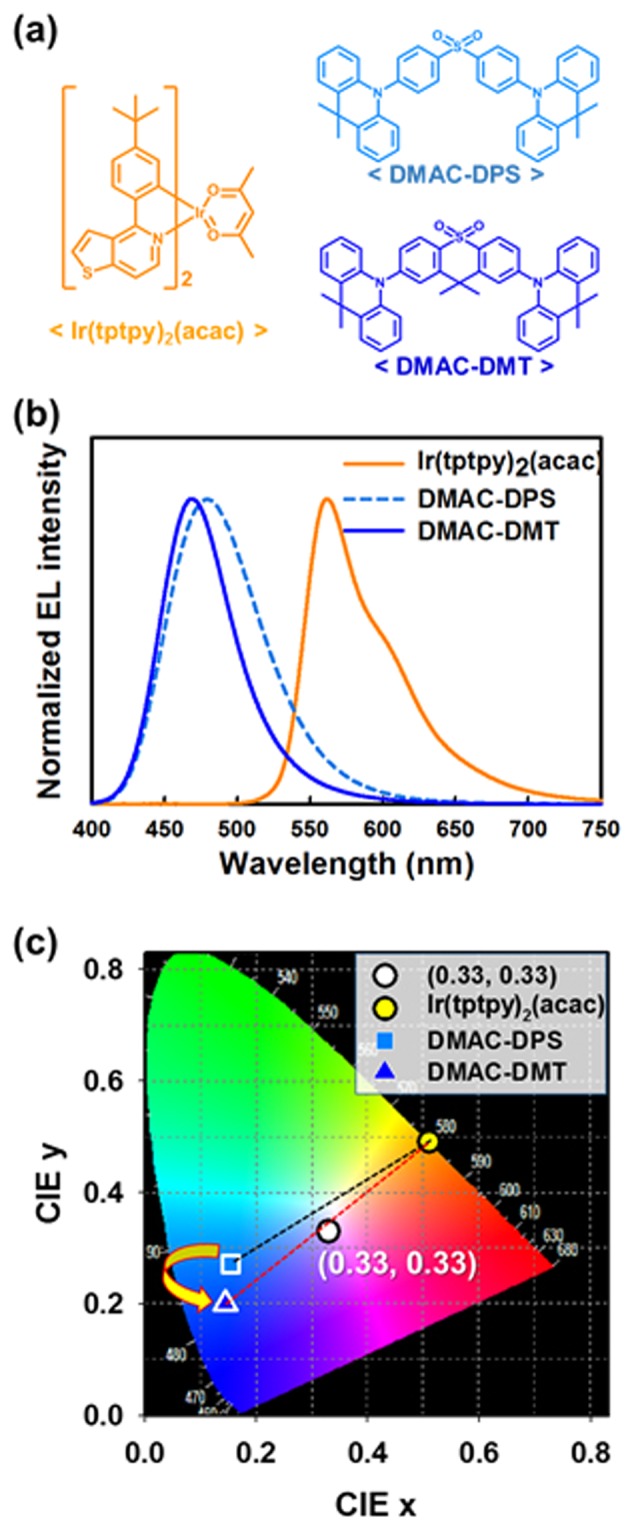
(**a**) Molecular structure of yellow phosphorescent emitter (Ir(tptpy)_2_(acac)) and blue TADF emitters (DMAC-DPS and DMAC-DMT), (**b**) EL spectra and, (**c**) CIE color coordinates of OLEDs using Ir(tptpy)_2_(acac), DMAC-DPS, and DMAC-DMT emitters.

CIE Color coordinates of blue and yellow emitters are very important parameter for making cool white emission. Thus, we carefully selected blue TADF emitter and yellow phosphorescent emitter. Herein, for the yellow phosphorescent emitter, Ir(tptpy)_2_(acac) is selected because of its pure yellow emission with almost 100% photoluminescence quantum yield (PLQY)^15,16^. As a high-efficiency blue TADF emitter, DMAC-DPS is the most famous and widely used material with very high quantum yield, but it has a sky-blue emission color due to broad emission spectrum resulting from rotational motion of phenyl units. Therefore, this emitter cannot be efficient for cool white emission in combination with the yellow emitter as shown in Fig. 2 ^17^. On the other hand, DMAC-DMT increases the rigidity of molecular structure compared with DMAC-DPS by interlocking the phenyl units, resulting in higher PLQY and deep blue color emission due to suppressed rotational motion of phenyl units^17^. Indeed, DMAC-DMT can make cool white emission in combination with the yellow emitter. Hence, we selected DMAC-DMT as a blue emitter for further evaluation.

### Design of blue and white OLED structure based on optical simulation

**Figure 3:**
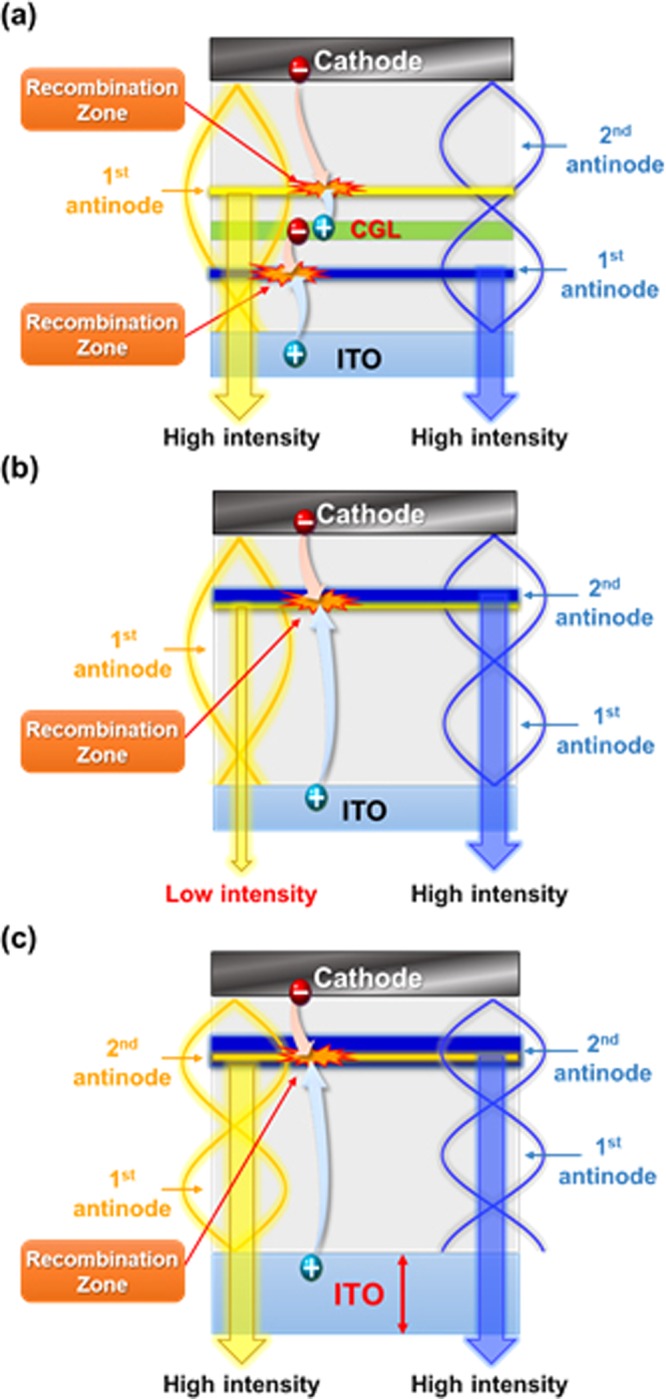
Schematic illustrations of antinode and EML positions for yellow and blue emissions in tandem and single-stack WOLEDs. (**a**) Tandem WOLED that easily fits both of blue and yellow EMLs to their respective antinode positions through spatially separated two recombination zones by charge generation layer (CGL), (**b**) Single-stack WOLED that cannot fit yellow EML to its antinode position, (**c**) Single-stack WOLED that fits both of blue and yellow EMLs to their antinode positions by changing interference condition of yellow and blue emissions in the WOLED through ITO thickness tuning.

To maximize the out-coupling efficiency of OLEDs, EMLs should be located at the antinode positions, wherein the electromagnetic waves generated from the emitter in the EML are constructively interfered^18,19^. Hence, it is very important in designing OLED structure to determine the device thickness (distance between transparent and reflective electrode) and the EML position by considering antinode condition of the EML emission spectrum. In particular, WOLED requires precise device engineering for high efficiency because two or more EMLs with different emission wavelengths are placed within the same device thickness. The antinode positions of the EMLs with different emission wavelengths are placed at different location for same device thickness condition. Unlike tandem WOLEDs, the difference in antinode position of the EML with respect to the emission wavelength makes the device design of single-stack WOLED more difficult. In the case of tandem structure with two EMLs (yellow and blue emission), blue EML and yellow EML can be easily placed in the respective antinode position because charge generation layer (CGL) generates two spatially separated recombination zones as shown in Fig. 3a. Whereas, single-stack structure without CGL makes just one recombination zone and it is difficult to find the recombination zone that satisfies both of the antinode positions of blue and yellow EMLs. A mismatch between the position of yellow antinode and recombination zone leads not only to reduction of EQE but also to unbalance EL intensity between yellow and blue emissions as shown in Fig. 3b. Hence, this problem can be resolved by finding an appropriate device thickness that approximates the location of the antinode of each EML within the total multiple EML thickness (25 nm) via changing the interference of electromagnetic wave inside the device. Indeed, this interference condition of both blue and yellow emissions can be obtained by tuning the thickness of transparent indium tin oxide (ITO) electrode as shown in Fig. 3c.

**Figure 4:**
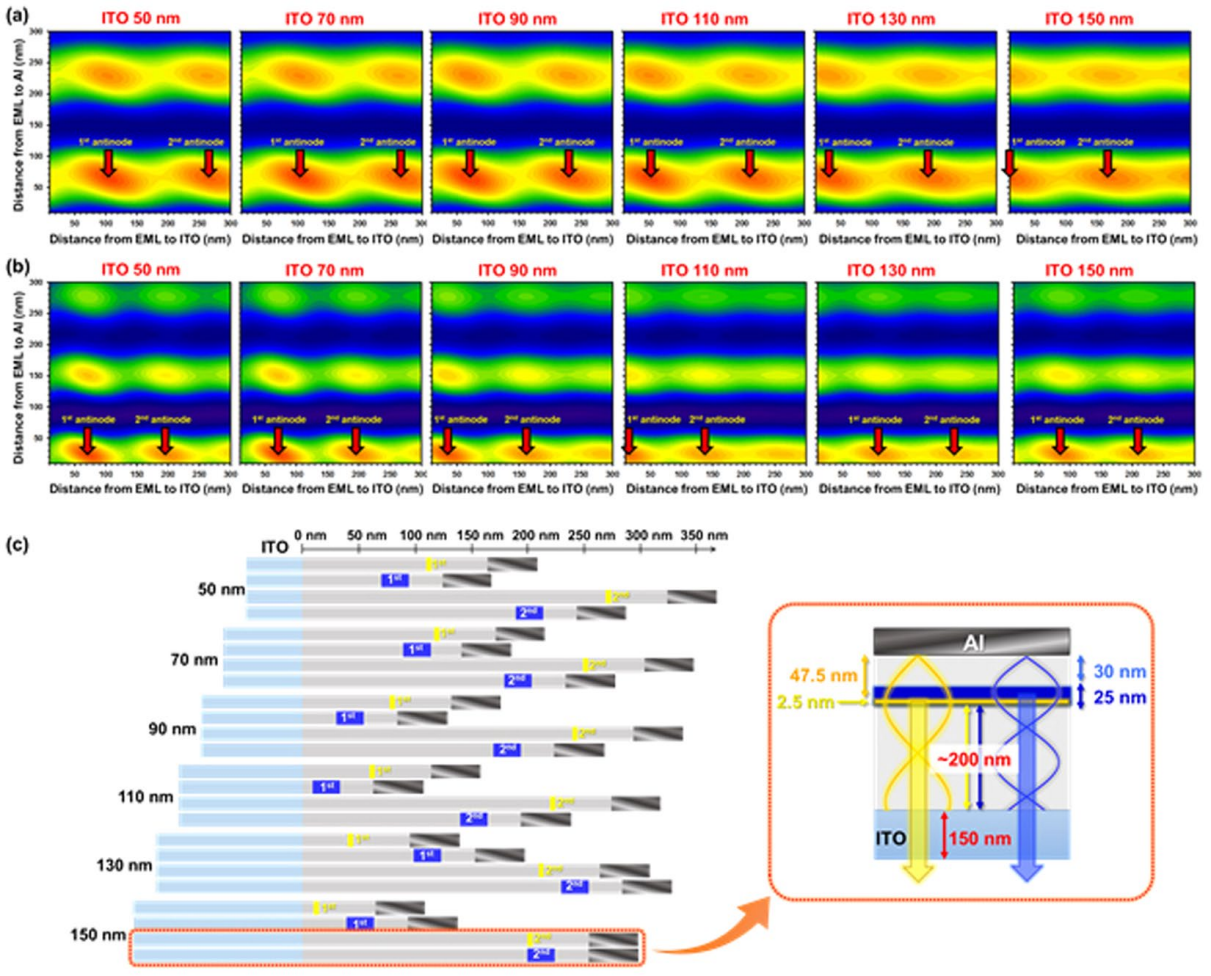
Contour plots for simulated radiance of (**a**) yellow emission from Ir(tptpy)_2_(acac), and (**b**) blue emission from DMAC-DMT as a function of the distance from EML to cathode (Al) and to anode (ITO), (**c**) Schematic diagrams of optimum thickness of yellow and blue devices at first and second antinode conditions depending on ITO thickness.

Fig. 4a,b shows calculated yellow (Ir(tytyp)_2_(acac)) and blue (DMAC-DMT) radiance as a function of the distance from EML to ITO and to reflective cathode (Al). Wherein, the thickness of yellow and blue EMLs is 2.5 and 25 nm, respectively. The distance from EML to Al (D_EML-Al_) greatly affects the change in radiance, and the D_EML-Al_ at the antinode position is independent on the ITO thickness variation. However, the distance from EML to ITO (D_EML-ITO_) weakly affects the change in radiance, and the D_EML-ITO_ at the antinode position is dependent on the ITO thickness variation. Therefore, it is necessary to find the accurate thickness of ITO that matches the D_EML-ITO_ at the blue and yellow antinode positions. Figure 4c visualizes optimum device conditions according to ITO thickness. Whereas, the D_EML-Al_ of yellow and blue devices were fixed to 30 and 47.5 nm, respectively, because thin yellow EML should be located in the blue EML to form blue-yellow-blue multi EML system. In the first antinode condition, the thickness of ITO that makes an equivalent D_EML-ITO_ for both yellow and blue devices was not found. On the other hand, in the second antinode position, 150 nm thick ITO demonstrated a similar D_EML-ITO_ (~200 nm) for blue and yellow devices. However, large thickness in both ITO and HTL may induce large ratios of waveguide modes and reduce light extraction. Hence, optical evaluation of devices with thin (70 nm) and thick (150 nm) ITO and different antinode conditions were performed. In the case of thin ITO (70 nm) and 1^st^ antinode condition, inappropriate emission pattern was observed due to strong emission from the yellow emitter. As a result, spectral ratio between blue and yellow was closer to the warm white emission (0.351, 0.410). In contrast, device with 150 nm thick ITO and 2^nd^ antinode condition showed cool white color emission (0.343, 0.350). Therefore, by considering the thickness condition shown in Fig. 4c (right side schematic), it is possible to maximize the out-coupling efficiency of yellow and blue light and balance yellow and blue light in single-stack WOLEDs.

### Device performance of blue TADF-OLEDs

**Figure 5:**
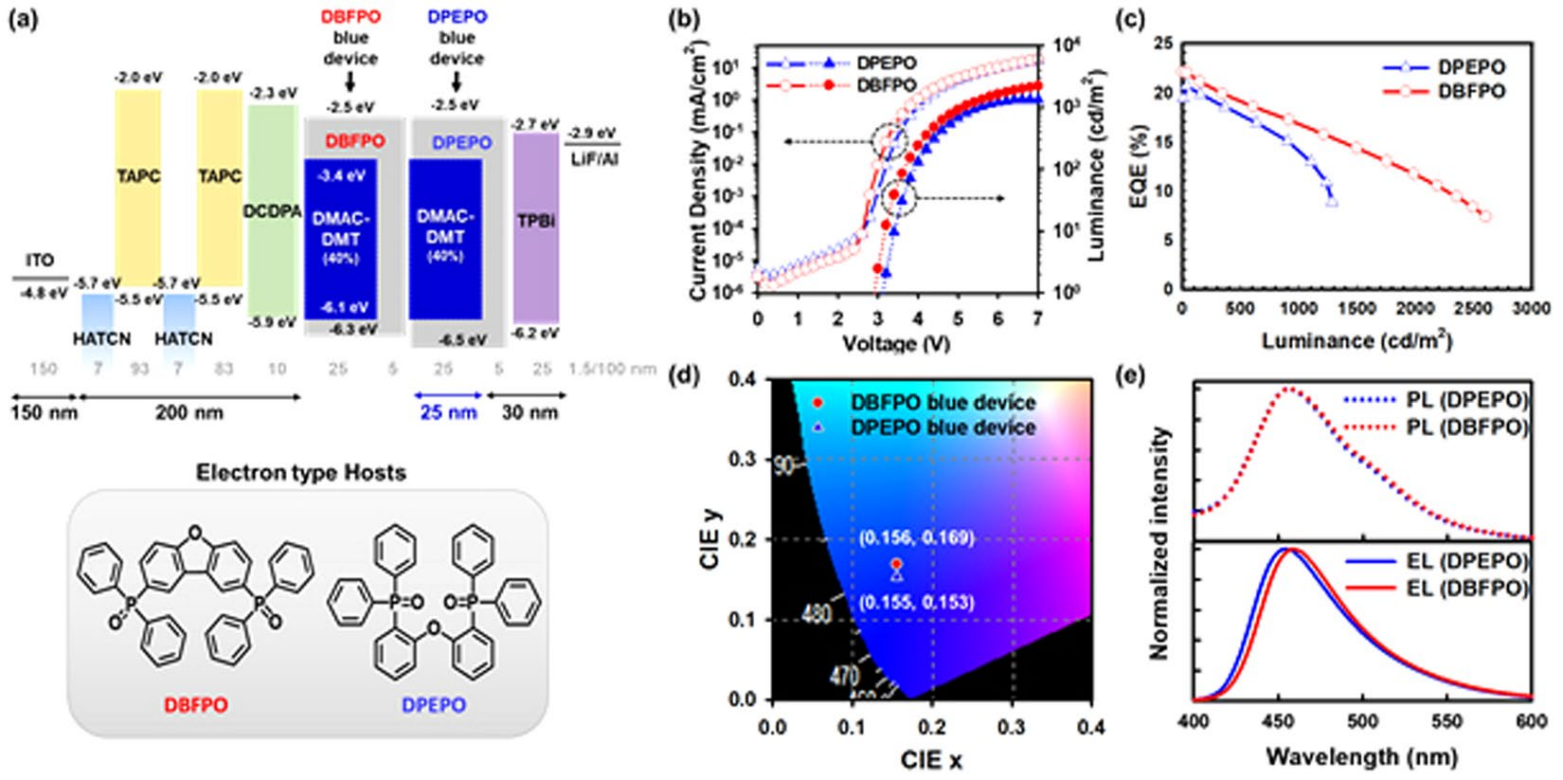
(**a**) Energy level diagram of blue TADF-OLED structure using DBFPO or DPEPO as a host and molecular structure of DBFPO and DPEPO, (**b**) current density versus voltage versus luminance curves, (**c**) EQE versus luminance curves, and (**d**) CIE color coordinates of DPEPO and DBFPO blue device, (**e**) Normalized PL spectra of DPEPO:DMAC-DMT(40%) and DBFPO:DMAC-DMT(40%) films and normalized EL spectra of DPEPO and DBFPO blue devices.

By considering an optical simulation results, the device structure was designed as shown in Fig. 5a. The fabricated device structure is as follows: ITO (150 nm)/HATCN (7 nm)/TAPC (93 nm)/HATCN (7 nm)/TAPC (83 nm)/DCDPA (10 nm)/Host:(40%) DMAC-DMT (25 nm)/Host (5 nm)/TPBI (25 nm)/LiF (1.5 nm)/Al (100 nm). To ensure the D_EML-Al_ for yellow EML that located between the blue EML, the recombination zone of blue TADF-OLED should be located near the HTL side. Thus, we selected DBFPO and DPEPO as host materials because their electron-transporting characteristics help to form the recombination near the HTL side, and also their higher triplet energy compared to that of DMAC-DMT prevents back energy transfer from the triplet of DMAC-DMT to the triplet of host. Prior to the evaluation of blue TADF-OLEDs, we optimized the doping concentration of DMAC-DMT in OLEDs. The device with 40% doped TADF emitter shows relatively higher luminance and EQE as well as good charge balance than those of other doping concentrations (Figure S1). Here, Fig. 5b–e shows device performances of blue TADF-OLEDs with DPEPO and DBFPO host materials. DBFPO and DPEPO based blue devices show not only very high EQEs of 22.1% and 20.7%, respectively, but also deep blue color coordinates of (0.156, 0.169) and (0.155, 0.153), respectively. Although PL spectra of DPEPO:DMAC-DMT(40%) and DBFPO:DMAC-DMT(40%) are almost identical, but EL spectrum of DBFPO based blue device is slightly red shifted compared to that of DPEPO comprised blue device. Likewise, current density is higher for DBFPO device than that of DPEPO at the same operating voltage. From Fig. 5b,e, we deduced that the recombination zone of DBFPO contained blue device is placed closer to the HTL side compared to that of DPEPO based blue device due to higher electron conductivity of DBFPO than DPEPO. In comparison to performances of DPEPO device, DBFPO comprised OLED is superior in EQE, efficiency roll-off characteristic, and operating voltage but it is inferior in color coordinates.

**Figure 6:**
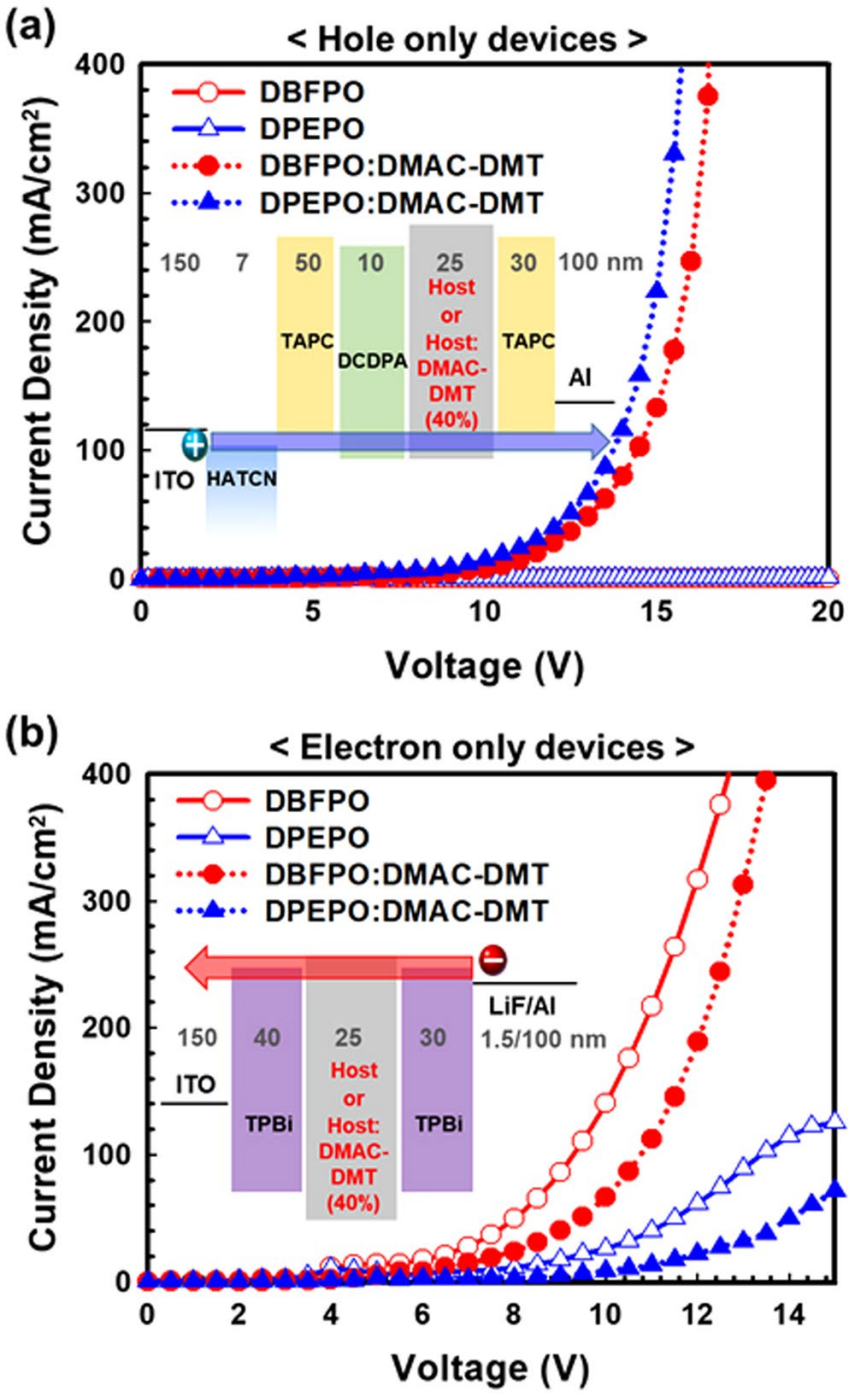
Current density versus voltage curves of (**a**) HODs and (**b**) EODs. Inset: device structure of HODs and EODs with host films (DBFPO (red open circle) or DPEPO (blue open triangle)) or EML films (DBFPO:40% DMAC-DMT (red filled circle) or DPEPO:40% DMAC-DMT (blue filled triangle)).

In order to investigate the basis cause of such different device characteristics for both host materials, we compared charge transporting and PL properties of the EMLs. To study hole- and electron-transporting properties of host and host:DMAC-DMT(40%) films, hole only device (HOD) and electron only device (EOD) were fabricated with the device structure shown in inset of Fig. 6a,b, respectively. As can be seen in current density versus voltage (J-V) curves of HODs (Fig. 6a), devices with neat DBFPO and DPEPO films does not show any hole transport, but DBFPO:DMAC-DMT and DPEPO:DMAC-DMT based HODs display substantial hole current. This demonstrates that both host materials have very poor hole conductivity and the hole current in the EML system mainly flows through DMAC-DMT. Although, significant hole current flows through DMAC-DMT in both HODs, the current density in DPEPO:DMAC-DMT comprised device is higher than that of DBFPO:DMAC-DMT. These results are attributed to hole-scattering effect of host materials. A shallower highest occupied molecular orbital (HOMO) level of DBFPO (−6.3 eV) (DPEPO (−6.5 eV)) reduces hole transport of DMAC-DMT in the EML by acting as shallow scatter^20^. On the other hand, in the case of J-V curves of EODs, the current densities of devices with pristine host film is higher than that of doped host and dopant system (host:DMAC-DMT based device), indicating that the electron conductivity of host materials is higher than the DMAC-DMT and additionally, DMAC-DMT is indeed acting as a deep electron trap, which leads to reduction of electron conductivity in the EMLs (host:DMAC-DMT). Furthermore, EOD with DBFPO has higher electron current density than that of DPEPO contained device despite of the same lowest unoccupied molecular orbital (LUMO) level of DBFPO and DPEPO, which signifies higher electron conductivity of DBFPO compared to that of DPEPO. It is important to note that, due to the higher electron conductivity of DBFPO and lower hole conductivity of DMAC-DMT in the EML of DBFPO based blue device, the recombination zone has formed close to the HTL interface than that of DPEPO device. Hence, this result provides the evidence of slightly red-shifted EL spectrum (Fig. 5e) and higher current density (Fig. 5b) in DBFPO based OLED compared with DPEPO contained device.

**Figure 7:**
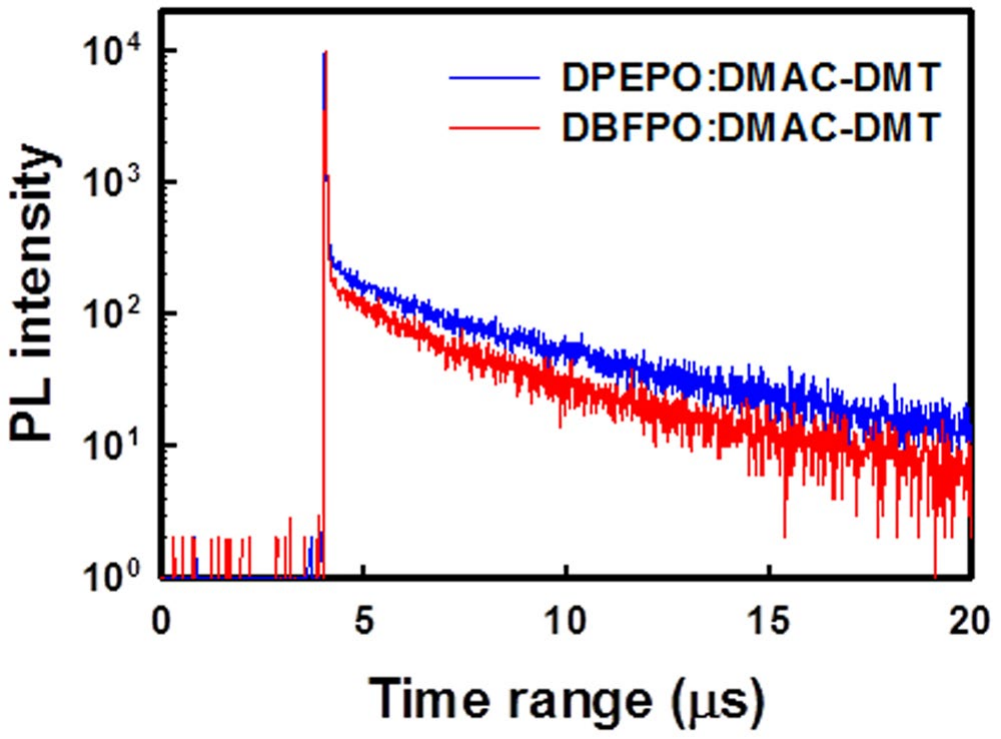
Transient PL decay curves of DPEPO:40% DMAC-DMT (blue line) and DBFPO:40% DMAC-DMT (red line).

To get the deep insight of OLED performances, photophysical properties of host:DMAC-DMT(40%) films are compared by measuring PLQY and transient PL, and their rate constants are also calculated using previously reported method as displayed in Table 1 and Fig. 7 ^21,22^. As can be seen, the transient PL decay characteristics of host:DMAC-DMT(40%) films are clearly distinguished by prompt and delayed components. The prompt and strong delayed components are originated from the fluorescence and TADF of DMAC-DMT, respectively. The exciton lifetime of the delayed component (τ_d_) of DBFPO:DMAC-DMT(40%) film (2.45 μs) is shorter than that of DPEPO:DMAC-DMT(40%) (3.88 μs) film. A shorter delayed exciton lifetime is beneficial for reducing triplet-triplet and triplet-polaron annihilation in the EML of OLED, which leads to suppression of efficiency roll-off characteristic^23–25^. This result indicates the main reason behind the lower efficiency roll-off characteristic of DBFPO device compared to that of DPEPO device (Fig. 5c). Moreover, by considering previous reports and assuming negligible (≈0) non-radiative decay rate from the singlet excited state, respective rate constants are evaluated for both studied films^21,22^. Here, DBFPO:DMAC-DMT(40%) film exhibits not only higher rate constant for RISC (k_RISC_) of about 1.6 × 10^6^ s^−1^ but also high PLQY of 91% compared to that of DPEPO:DMAC-DMT(40%) (1.0 × 10^6^ s^−1^ and 78%), which results in high EQE for DBFPO contained blue device than the DPEPO based blue device.

**Table 1:** Photophysical properties of DPEPO:DMAC-DMT(40%) and DBFPO:DMAC-DMT(40%) films.

EML film	*ϕ*_PL_ (%)	*ϕ*_p_ (%)	*ϕ*_d_ (%)	*τ*_p_ (ns)	*τ*_d_ (us)	*κ*_p_ (s^−1^)	*κ*_d_ (s^−1^)	*κ*^S^_r_ (s^−1^)	*κ*_ISC_ (s^−1^)	*κ*_RISC_ (s^−1^)	*κ*^T^_nr_ (s^−1^)
DPEPO:DMAC-DMT(40%)	78	18	78	22.52	3.88	4.4 × 10^7^	2.6 × 10^5^	8.0 × 10^6^	3.6 × 10^7^	1.0 × 10^6^	6.9 × 10^4^
DBFPO:DMAC-DMT(40%)	91	22	68	22.70	2.45	4.4 × 10^7^	4.1 × 10^5^	9.7 × 10^6^	3.4 × 10^7^	1.6 × 10^6^	5.2 × 10^4^

*ϕ*_PL_: photoluminescence quantum yield, *ϕ*_p_: quantum yield of prompt component, *ϕ*_d_: quantum yield of delayed component, *τ*_p_: exciton lifetime of prompt component, *τ*_p_: exciton lifetime of delayed component, *κ*_p_: rate constant of prompt emission, *κ*_d_: rate constant of delayed emission, *κ*^S^_r_: rate constant of radiative decay of singlet exciton, *κ*_ISC_: rate constant of intersystem crossing (ISC), *κ*_RISC_: rate constant of reverse ISC (RISC) from triplet state to singlet state, *κ*^T^_nr_: rate constant of non-radiative decay of triplet exciton.

### Device performance of single-stack hybrid WOLEDs

**Figure 8:**
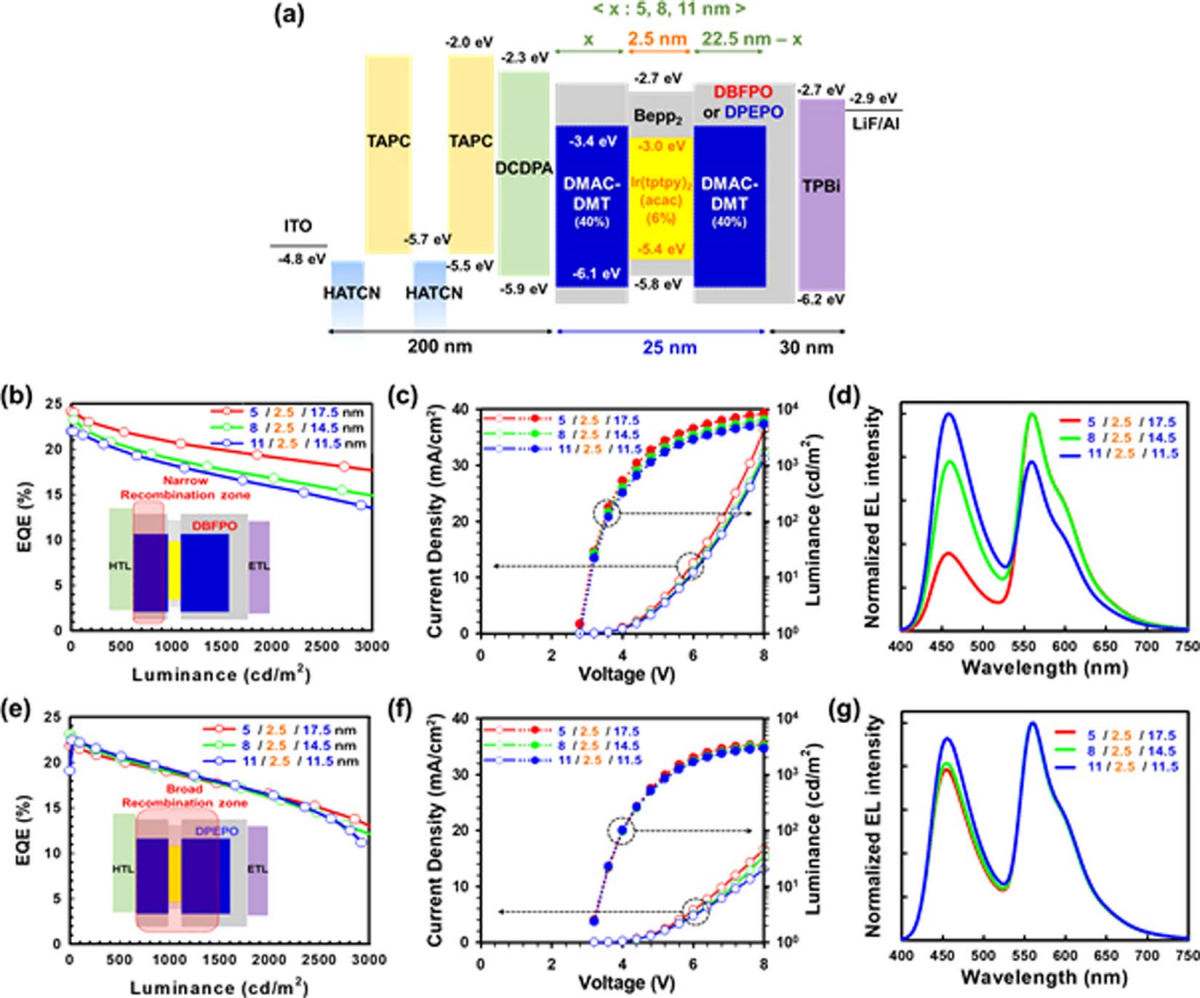
(**a**) Energy level diagram with detail device structure of single-stack hybrid WOLEDs. Wherein, the thin yellow EML has three positions with different distance (x: 5, 8, and 11 nm) from HTL. (**b**) EQE-luminance curves of DBFPO and (**e**) DPEPO based white devices with different x value. Inset: Schematic diagram shows recombination zone in the blue-yellow-blue multiple EML system of respective white devices. J-V-L curves and normalized EL spectra of DBFPO (**c**,**d**), and DPEPO (**f**,**g**) comprised white devices.

**Figure 9:**
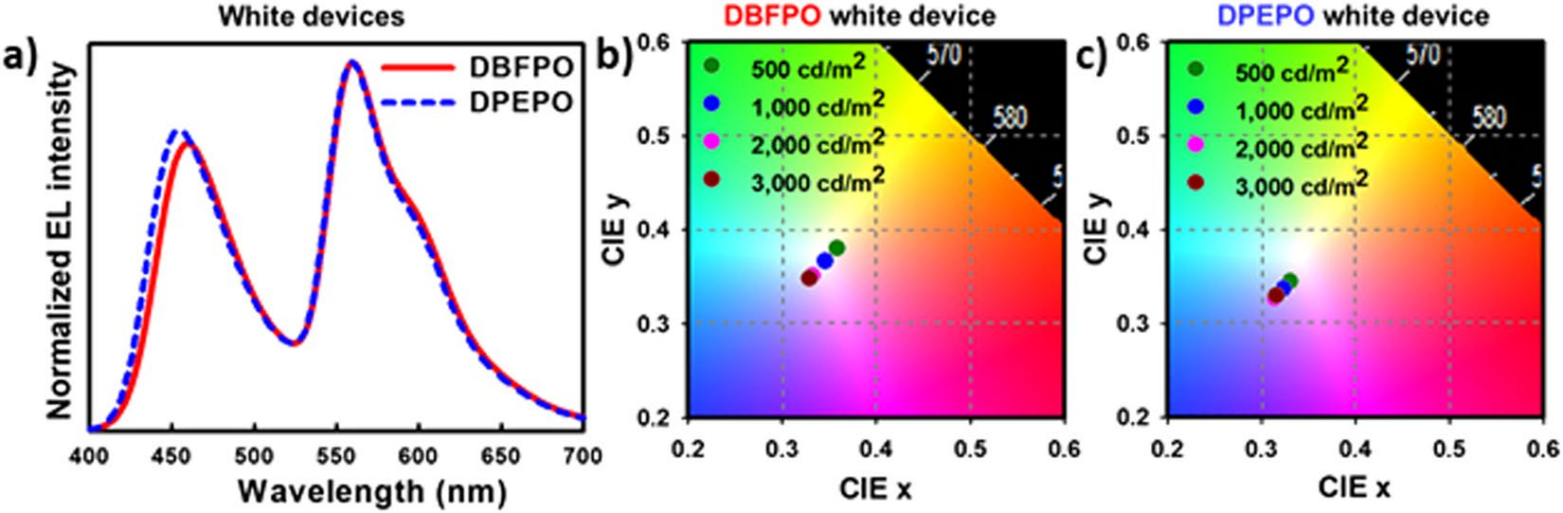
(**a**) Comparison of normalized EL spectra of DPEPO and DBFPO based white devices with blue-yellow-blue EML thickness of 8/2.5/14.5 nm at 1000 cd/m^2^, (**b**) Change in color coordinates of the DBFPO and (**c**) DPEPO contained white device depending on luminance.

By considering the position of recombination zone and high EQE of DBFPO based blue device, it may seem that DBFPO is more suitable for blue host material in high performance WOLED device. More interestingly, the use of DBFPO as a host also allows us to place thin yellow EML close to the HTL side; thereby the distance between yellow EML and Al for the antinode position of yellow emission can be obtained. On the other hand, DPEPO host has an advantage in color coordinate characteristics. To investigate the effect of host materials in blue-yellow-blue multiple EML system and to achieve cool white emission, single-stack hybrid WOLED (Fig. 8a) was fabricated with following configuration: ITO (150 nm)/HATCN (7 nm)/TAPC (93 nm)/HATCN (7 nm)/TAPC (83 nm)/DCDPA (10 nm)/Host:(40%) DMAC-DMT (x nm)/Bepp_2_:(6%) Ir(tptpy)_2_(acac) (2.5 nm)/Host:(40%) DMAC-DMT (22.5 - x nm)/Host (5 nm)/TPBI (25 nm)/LiF (1.5 nm)/Al (100 nm). Herein, the thin yellow EML position has varied to adjust the intensity ratio of yellow and blue emission. As shown in Table 2 and Fig. 8b,d, WOLED with DBFPO host exhibits large changes in EQE and EL spectra depending on the position of yellow EML. As distance between yellow EML and HTL (DCDPA) increases, EQE and EL intensity of yellow emission are reduced. This result can be attributed to narrow recombination zone formed in the front blue EML of blue-yellow-blue system due to high electron conductivity of DBFPO and low hole conductivity of DMAC-DMT as depicted in the inset of Fig. 8b, which suggest that the excitons generated in the yellow EML by the triplet exciton diffusion and Dexter energy transfer are reduced with respect to increase in distance between the HTL/blue EML interface and yellow EML. Contrary to our expectation, the recombination zone biased towards the HTL side makes it difficult to locate the yellow EML near the HTL. Indeed, when the yellow EML is located close to the HTL side, too much excitons are generated in the yellow EML, which leads to warm white emission basically due to high exciton density in the narrow recombination zone near the HTL side. Furthermore, the narrow recombination zone biased towards the HTL not only causes a red-shift in the blue emission (which makes it difficult to obtain CIE color coordinates of 0.33, 0.33 in combination with the yellow emission), but also lowers the color stability according to luminance as shown in Fig. 9a,b. Therefore, DBFPO is not suitable for blue host in the blue-yellow-blue multiple EML system for cool white emission.

**Table 2:** Device characteristics of single-stack hybrid white OLEDs utilizing DBFPO or DPEPO as a blue host and three different yellow EML positions.

White device		EQE (%)	CIE Color coordinate (x, y)	Turn-on/operating voltage (V)	Power efficiency (lm/W)	CRI	CCT (K)
Blue host	Blue/yellow/blue EML thickness (nm)	Max/1000 nit	1000 nit	1nit/1000 nit	Max/1000 nit	1000 nit	1000 nit
DBFPO	5/2.5/22.5	24.2/20.6	0.394, 0.414	2.4/4.3	72.9/35.5	49	3929
	8/2.5/14.5	23.2/19.4	0.346 0.367	2.4/4.6	56.7 /30.7	62	5446
	11/2.5/11.5	22.0/17.9	0.283, 0.300	2.4/4.7	56.7 /22.2	72	8997
DPEPO	5/2.5/22.5	21.8/19.0	0.329, 0.341	2.6/5.2	54.5 /24.3	62	5722
	8/2.5/14.5	23.1/19.4	0.324, 0.337	2.6/5.3	59.0 /24.6	63	5893
	11/2.5/11.5	22.4/19.7	0.316, 0.326	2.6/5.3	49.0 /24.2	66	6360

On the other hand, due to relatively lower electron conductivity of DPEPO and higher hole mobility of DMAC-DMT in DPEPO medium, the recombination zone can be broadly formed over the whole blue-yellow-blue EMLs of DPEPO comprised WOLED as depicted in the inset of Fig. 8e. As a result, reduced change in EQE and EL spectra depending on the yellow EML position as shown in Table 2 and Fig. 8e,g. In addition, the broad recombination zone generates hypsochromic shift in the blue emission to obtain cool white emission and also improve color stability depending on luminance as demonstrated in Fig. 9a,c. Consequently, by using an optimum blue-yellow-blue (8/2.5/14.5 nm) multiple EML system in DPEPO contained WOLED, very high EQE of 23.1% and cool white emission with CIE color coordinates of 0.324, 0.337 are attained. To the best of our knowledge, this is the best device performances reported to date for hybrid CWOLED. Furthermore, color rendering index (CRI) and correlated color temperature (CCT) are the crucial parameters in WOLED; hence we evaluated the CRI and CCT values of all fabricated single-stack hybrid WOLEDs. The CRI and CCT values of WOLEDs with DBFPO and DPEPO are summarized in Table 2. Similarly, the power efficiency versus luminance characteristics of WOLEDs and their respective values at 1000 cd/m^2^ are shown in Fig. S3 and Table 2.

## Conclusion

In conclusion, single-stack CWOLED with blue-yellow-blue hybrid multiple EML structure was developed by utilizing blue TADF and yellow phosphorescent emitters. The device structure was optimized to achieve balanced and highly efficient blue and yellow emission by using constructive interference condition of the blue and yellow emission as well as exciton dynamics in the multiple EML system. Both of constructive interference condition for yellow and blue emissions are satisfied even in the single-stack OLED using 150 nm thick ITO and second antinode condition, which results in high out-coupling efficiency and very high EQE of 23.1%. The well-balanced intensities of blue and yellow emissions are attained in the multiple EML system by controlling recombination zone and utilizing host materials with different electron conductivity, thereby cool white emission (0.324, 0.337) with high color stability depending on luminance are realized. These results demonstrated that even single-stack hybrid CWOLEDs can achieve high efficiency comparable to those of tandem hybrid CWOLEDs. This work provides an approach to obtain highly efficient cool white emission from single-stack hybrid WOLED and increases the feasibility of single-stack hybrid WOLED for large area and high-resolution display applications. Additionally, we anticipate that our approach can be realized for RGB three-component WOLED either by doping ultra-low concentration of red phosphorescent emitter in yellow EML or by inserting separate ultra-thin red EML.

## Experimental Section

### Materials

3,5-Di(9H-carbazol-9-yl)-N,N-diphenylaniline (DCDPA), bis[2-(diphenylphosphino) phenyl] ether oxide (DPEPO), 2,8-bis(diphenylphosphine oxide) dibenzofuran (DBFPO), and 2,7-bis(9,9-dimethylacridin-10(9H)-yl)-9,9-dimethyl-9H-thioxanthene 10,10-dioxide (DMAC-DMT) were synthesized according to the previously reported methods^17,26–28^. The other materials such as 1,4,5,8,9,11-hexaazatriphenylene-hexacarbonitrile (HATCN), 1,1-bis[(di-4-tolylamino) phenyl] cyclo-hexane (TAPC), Bis(2-(2-hydroxyphenyl)pyridinato)beryllium (Bepp_2_), Acetylacetonatobis(4-(4-tert-butylphenyl)-thieno[3,2-c]pyridinato-C2,N)iridium (Ir(tptpy)_2_(acac)), 2,2′,2″-(1,3,5-benzinetriyl)-tris(1-phenyl-1-H-benzimidazole) (TPBi), lithium fluoride (LiF), and aluminum (Al) were purchased from EM Index, Jilin OLED Material Tech Co., Ltd., and Luminescence Technology Corp., respectively. The DCDPA was used as an exciton-blocking layer. HATCN and TAPC were incorporated as hole-injecting and hole-transporting layer, respectively. DBFPO and DPEPO were used as blue TADF host and DMAC-DMT was used as blue TADF emitter. For the yellow EML, Ir(tptpy)_2_(acac) and Bepp_2_ were used as yellow emitter and yellow host, respectively. TPBi was used as electron-transporting layer.

### Device simulations

The extensive optical simulations of yellow and blue OLEDs were performed using semiconducting emissive thin film optics simulator (SETFOS 4.1). The n and k values of studied organic materials were obtained from the spectroscopic ellipsometry measurements.

### Device fabrication

The ITO glass substrates with active area of 4.0 mm^2^ were sequentially cleaned by using ultrasonic cleaner in acetone, isopropyl alcohol, and deionized water for 10 minutes each. All the organic and inorganic layers were evaporated on the pre-cleaned ITO glass substrates in thermal evaporator under high vacuum pressure of 10^−7^ Torr. After the deposition process, complete device was encapsulated by using desiccant attached glass cover and UV-cured resin under nitrogen atmosphere.

### Characterization of device and material

The current density-voltage-luminance (J-V-L) curves, Commission Internationale de l’Eclairage (CIE) 1931 color coordinate, and electroluminescence (EL) spectra were obtained by using a spectroradiometer (CS-2000, Konica Minolta). Photoluminescence (PL) spectra of 50 nm-thick EML films (DBFPO:DMAC-DMT(40%) and DPEPO:DMAC-DMT(40%)) were measured using fluorescence spectrophotometer (Hitachi F-7000) at room temperature. The photoluminescence quantum yields (PLQY) of the EML films were obtained from fluorescence spectrometer (JASCO FP-8500) installed with an integrated sphere. The transient PL decay curves of the EML films were recorded from Quantaurus-Tau fluorescence lifetime measurement system (C11367-03, Hamamatsu Photonics Co.) under N_2_ atmosphere at room temperature. The exciton lifetimes of prompt and delayed components of the PL decay curves were calculated using single and triple-exponential decaying functions^22^.

## Electronic supplementary material

Supplementary Information
