# Supplementary material for: Highly efficient single-stack hybrid cool white OLED utilizing blue thermally activated delayed fluorescent and yellow phosphorescent emitters
Source: Sci Rep. 2018 Nov 2;8:16263. doi: 10.1038/s41598-018-34593-3 (PMC6214941; doi:10.1038/s41598-018-34593-3)
Supplement: Supplementary file 1 — Supplementary Information [file 41598_2018_34593_MOESM1_ESM.docx]

Supporting Information

Highly efficient single-stack hybrid cool white OLED utilizing blue thermally activated delayed fluorescent and yellow phosphorescent emitters

Gyeong Woo Kim, Hyeong Woo Bae, Raju Lampande, Ik Jang Ko, Jin Hwan Park, Chae Young Lee and Jang Hyuk Kwon*

# Department of Information Display, Kyung Hee University, Dongdaemoon-gu, Seoul 130-701, Republic of Korea


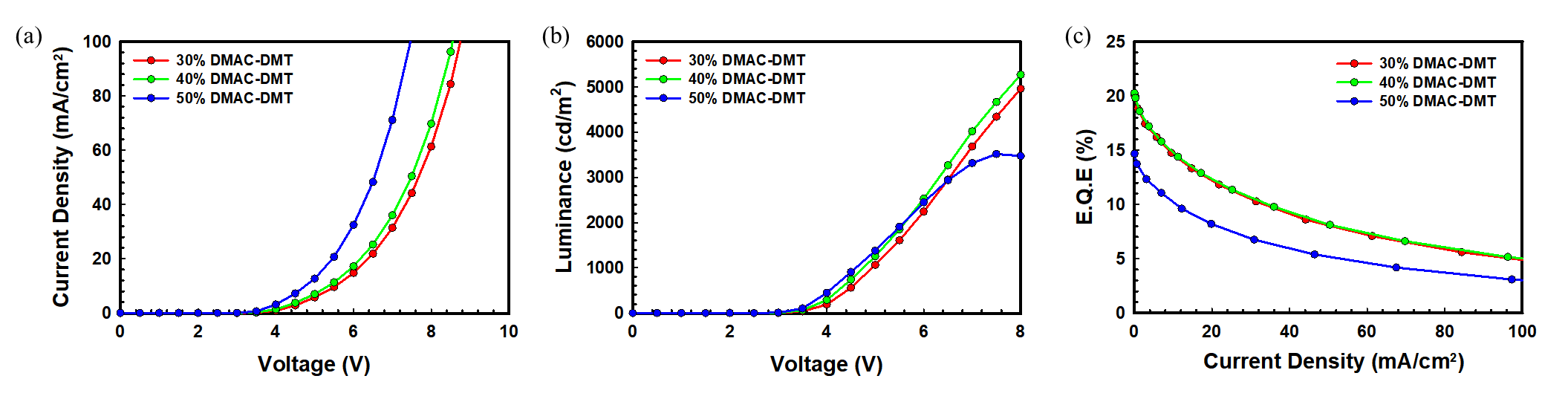


Figure S1. Device performances of blue OLED devices with different doping concentration (30 %, 40 % and

50 %) of DMAC-DMT. a) current density versus voltage, b) luminance versus voltage, and c) external quantum

efficiency versus current density.

Figure S2. Power efficiency versus luminance characteristics of blue OLED devices with DPEPO and DBFPO host materials.


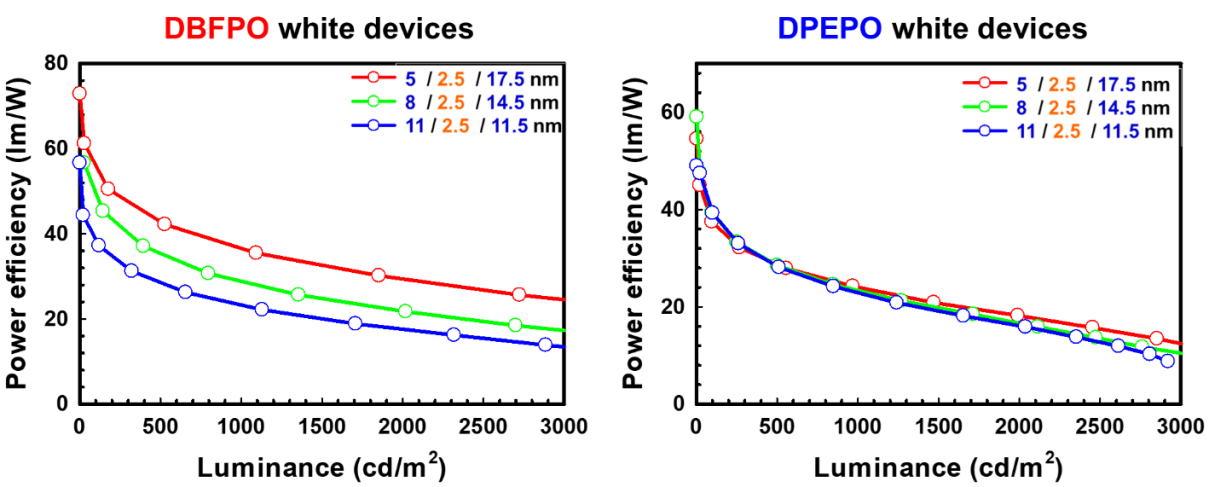


Figure S3. Power efficiency versus luminance characteristics of WOLEDs with DBFPO and DPEPO host materials.
